# Krill Excretion Boosts Microbial Activity in the Southern Ocean

**DOI:** 10.1371/journal.pone.0089391

**Published:** 2014-02-19

**Authors:** Javier Arístegui, Carlos M. Duarte, Isabel Reche, Juan L. Gómez-Pinchetti

**Affiliations:** 1 Instituto de Oceanografía y Cambio Global (IOCAG), Universidad de Las Palmas de Gran Canaria, Las Palmas de Gran Canaria, Spain; 2 Instituto Mediterráneo de Estudios Avanzados (IMEDEA), CSIC-UIB, Esporles, Illes Balears, Spain; 3 The UWA Oceans Institute, The University of Western Australia, Perth, Australia; 4 Departmento de Ecología, Facultad de Ciencias, Universidad de Granada, Granada, Spain; 5 Spanish Bank of Algae (BEA), Telde, Gran Canaria, Spain; University of Tasmania, Australia

## Abstract

Antarctic krill are known to release large amounts of inorganic and organic nutrients to the water column. Here we test the role of krill excretion of dissolved products in stimulating heterotrophic bacteria on the basis of three experiments where ammonium and organic excretory products released by krill were added to bacterial assemblages, free of grazers. Our results demonstrate that the addition of krill excretion products (but not of ammonium alone), at levels expected in krill swarms, greatly stimulates bacteria resulting in an order-of-magnitude increase in growth and production. Furthermore, they suggest that bacterial growth rate in the Southern Ocean is suppressed well below their potential by resource limitation. Enhanced bacterial activity in the presence of krill, which are major sources of DOC in the Southern Ocean, would further increase recycling processes associated with krill activity, resulting in highly efficient krill-bacterial recycling that should be conducive to stimulating periods of high primary productivity in the Southern Ocean.

## Introduction

Antarctic krill (*Euphasia superba*), with a biomass estimated at 379 million tonnes [1], is one of the most abundant animals on Earth and the central node of the Antarctic food web, supporting the large biomass of megafauna characteristic of this ecosystem [2], [3]. The large biomass of krill is maintained by intense grazing not only on phytoplankton, but also on other planktonic organisms, including protists and copepods [4], [5], [6]. Smetacek [7] hypothesized that krill activity may also stimulate phytoplankton growth, thereby conditioning the ecosystem to maintain high productivity. Tovar-Sánchez et al. [8] provided additional evidence for this notion by showing that krill release large amounts of limiting elements, including Fe, P and N, in the Southern Ocean, thereby creating the conditions to support subsequent algal blooms.

The emerging view of krill as efficient recyclers focuses, so far, on feedbacks between krill and primary producers, but does not address the role of krill activity on the micro-heterotrophic community. Bacteria in the Southern Ocean are considered to be strongly limited by the availability of labile DOC [9], [10], [11], imposing low growth rates that render them vulnerable to top-down control by consumers [12]. Recent evidence shows that in addition to releasing large amounts of inorganic nutrients [8], krill are also an important source of organic materials to the water column, both particulate (faecal pellets), which may sink out of the mixed layer rapidly [13], [14], [15], and dissolved [16], [17]. The release of large amounts of dissolved organic matter by krill activity may also stimulate bacterial communities in the Southern Ocean, further contributing to the role of krill in accelerating recycling processes in the ecosystem.

Here we test the role of krill excretion of dissolved products in stimulating heterotrophic bacteria in the Southern Ocean. We do so on the basis of three experiments where dissolved excretory products released by krill were added to a bacterial assemblage, free of grazers. The response of bacteria, in terms of biomass, production, oxygen consumption and growth, was compared to that in treatments receiving comparable levels of ammonium to those added with the krill excretory products, and controls with no added substrates.

## Materials and Methods

### Study Area

**Figure 1:**
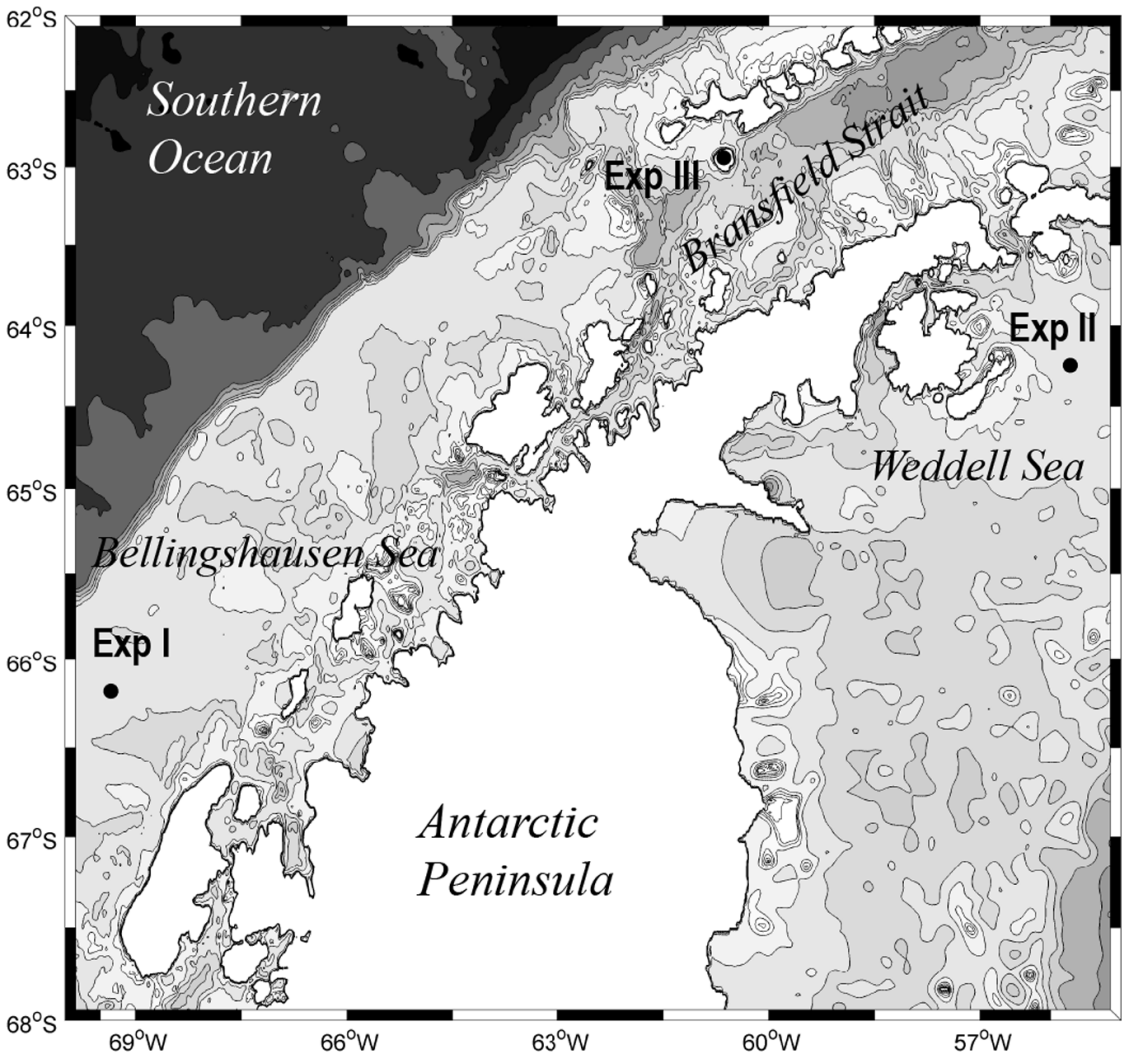
Map of stations. Location of the stations from where water was collected for experiments I, II and III.

The samples for the experiments were obtained from 5 m depth through a clean seawater pump, at three stations with different oceanographic conditions, along the Antarctic Peninsula sector of the Southern Ocean (Fig. 1), during the ICEPOS 2005 cruise (3–17 February, 2005) on board the R/V Hespérides. No specific permissions were required to collect water from the stations, and the field studies did not involve endangered or protected species. The station where experiment I was initiated was located south of the Polar Circle in the Bellingshausen Sea (−66.18°S, −69.35°W); the station for experiment II in the western Weddell Sea (−64.25°S, −55.72°W); and the station for experiment III in the Bransfield Strait, south of Deception Island (−62.94°S, −60.64°W). The three stations varied in their physical and biological properties (Table 1), with that in the Weddell Sea placed in the region with the coolest temperatures (<0°C) but highest bacterial production (184 ng C l^−1^ h^−1^).

**Table 1:** Comparative analysis of biological variables *in situ* and at the onset of experiments.

In situ	Bellingshausen Sea	Weddell Sea	Bransfield Strait
**Temperature** (°C)	1.29	–0.49	1.47
**Salinity**	33.5	33.9	33.9
**NH_4_^+^** (µM)	0.44 (0.10)	0.54 (0.25)	0.18 (0.04)
**DOC** (µM)	55.9 (2.84)	58.2 (1.55)	51.1 (3.30)
**Fe** (nM)	0.55 (0.49)	0.13 (0.03)	0.62 (-)
**Chl a** (µg l^−1^)	0.54 (0.30)	3.17 (1.36)	4.68 (0.14)
**BA** (x10^5^ cell ml^−1^)	4.26 (1.05)	5.82 (1.92)	31.5 (11.5)
**BP-Leu** (ng C l^−1^ h^−1^)	4.83 (0.46)	184 (17.9)	23.1 (0.56)
**Experiments**	**I**	**II**	**III**
**NH_4_^+^ -T0 C** (µM)	0.45 (0.07)	1.22 (0.24)	0.45 (0.43)
**NH_4_^+^ -T0 A** (µM)	5.01 (0.11)	5.06 (0.12)	4.81 (0.17)
**NH_4_^+^ -T0 K** (µM)	11.7 (0.01)	2.56 (0.37)	4.01 (0.16)
**DOC-T0 C** (µM)	69.0 (8.09)	72.2 (4.32)	74.7 (3.13)
**DOC-T0 A** (µM)	71.8 (4.38)	69.4 (1.61)	75.2 (3.72)
**DOC-T0 K** (µM)	560 (28.3)	205 (21.2)	188 (45.3)
**Fe** (nM)	5.9 (−)	2.8 (−)	15.1 (−)
**BA-T0 C** (x10^5^ cell ml^−1^)	8.46 (1.28)	5.82 (0.42)	26.3 (1.80)
**BA-T0 A** (x10^5^ cell ml^−1^)	7.36 (1.94)	4.94 (1.12)	25.4 (3.16)
**BA-T0 K** (x10^5^ cell ml^−1^)	5.14 (0.47)	4.81 (0.51)	28.8 (1.41)
**BP-Leu-T0 C** (ng C l^−1^ h^−1^)	2.59 (0.31)	170 (3.38)	39.9 (0.58)
**BP-Leu-T0 A** (ng C l^−1^ h^−1^)	2.56 (0.65)	164 (8.10)	33.4 (0.42)
**BP-Leu-T0 K** (ng C l^−1^ h^−1^)	2.42 (0.23)	104 (3.55)	26.7 (0.25)

Upper panel: Physical and biological parameters at 5 m depth, from the three sampling sites (*in situ*) where water was collected for experiments. Mean and standard deviation values (of 2–3 replicates, in parenthesis) of ammonium (NH_4_^+^), dissolved organic carbon (DOC), iron (Fe), chlorophyll a (Chl a), bacterial abundance (BA) and bacterial production from Leucine uptake (BP-Leu). Lower panel: Mean and standard deviation values (of 2–3 replicates, in parenthesis) of NH_4_^+^, DOC, BA and BP at the onset (T0) of the three experiments in the two treatments (ammonium, A; krill excretion products, K) and controls (C). Fe = Iron concentrations in the pre-filtered water used for experiments, before adding any treatment.

### Experimental Design

The effect of krill excretion products on heterotrophic bacteria was tested using re-growth experiments. The bacterial assemblages used in the experiments were prepared by filtering surface seawater through a 5 µm and then 1.2 µm Millipore cartridge. A solution rich in krill excretion products was obtained by placing 20–30 freshly collected adult krill individuals, sampled using an IKMT net, as described in Tovar-Sánchez et al. [8], in a bucket containing 20 L of filtered (0.2 µm) surface seawater. The bucket containing the krill was placed in the dark in a temperature-controlled chamber (0.3±0.2°C) and ammonium concentrations were monitored daily until concentrations exceeded 30 µmol NH_4_^+^ L^−1^. The additions of krill excretion products, pre-filtered through 0.2 µm to avoid particles or bacteria growing in the bucket, were scaled to achieve a final concentration of 11.7, 2.5 and 4 µmol NH_4_ L^−1^ in experiments I to III, respectively. These concentrations would be in the range of those expected after 2 to 24 h of excretion of krill swarms, ranging in density from 1000 to 2000 individuals m^−3^ [18], [19], assuming an excretion rate of 200–300 nmol NH_4_^+^ ind^−1^ h^−1^ [20], [21] (i.e. 0. 4 to 14 µmol NH_4_ L^−1^).

In addition to a treatment receiving krill excretion products, a control – with no additions – was run in parallel, and a treatment with ammonium additions, added as ClNH_4_ to a final concentration of 5 µmol NH_4_^+^ L^−1^ in experiments I to III to test for the role of ammonium in controlling bacterial growth in the Southern Ocean. This concentration represented an approximate average value of the ammonium additions in the three krill treatments.

The pre-filtered (50 L) water from each of the initial treatments (control,+NH_4_^+^, and+krill excretion) was homogenously distributed into forty-two 125-mL borosilicate bottles (7 replicate bottles for each of the 6 sampling times), which were kept immersed in a bath at 0.3±0.2°C during incubations in the dark. The experiments lasted between 140 and 160 hours, during which samples were collected (in 24–36 hours intervals) for determinations of ammonium, oxygen concentration, and bacterial abundance.

### Samples Analyses

Ammonium (NH_4_^+^) and dissolved oxygen (DO) were measured at every time point during the incubation for all experiments. NH_4_^+^ was analyzed spectrofluorimetrically (with a Perkin Elmer LS-50B equipment) within 2 h of sample collection [22]. DO was measured in 5 of the 7 replicates per sample by the micro-Winkler technique, with colorimetric end-point detection, as described in [23]. The CV of replicated analyses was <0.05%.

Bacterial abundances (BA) were counted on board by flow cytometry using a FACScalibur system (Becton and Dickinson) with a 15 mW, 488 nm argon laser. Duplicate samples (4 ml) were fixed with 2% final concentration of paraformaldehyde, left for 15–30 min at 4°C and then stored frozen in liquid nitrogen until analyzed. Prior to analysis, 200 µl were stained with a DMSO-diluted SYTO-13 (Molecular Probes Inc.) stock (10∶1) at 2.5 µM final concentration. Bacterial biomass (BB) was estimated by inferring the cell biovolume from an experimentally derived relationship between green cell fluorescence (FL1) and cell biovolume: Biovolume (µm^3^) = 0.068+0.11 FL1; r^2^ = 0.66 [24]. BB was calculated assuming a cellular carbon content of 12 fg C cell^−1^ [25].

Integrated bacterial production (BP) was calculated from changes in biomass over consecutive times along the experiment. Bacterial respiration (BR) was estimated as the difference in DO between two consecutive sampling times. A respiratory quotient (RQ) = 1 was used to convert oxygen to carbon units. Bacterial carbon demand (BCD: BP+BR) and bacterial growth efficiency (BGE: BP/(BP+BR)) were calculated from BP and BR estimates (Table 2).

**Table 2:** Bacterial metabolism in experiments.

Experiment	BP	BR	BCD	BGE	NGR
	(µgC l^−1^ d^−1^)	(µgC l^−1^ d^−1^)	(µgC l^−1^ d^−1^)		(d^−1^)
**Bellingshausen (I)**					
Control	0.1	0.3	0.4	0.25	0.05
NH_4_^+^	0.3	3.4	3.7	0.07	0.12
Krill	97	583	680	0.14	0.97
**Weddell (II)**					
Control	2.7	3.4	6.1	0.44	0.36
NH_4_^+^	3.1	3.7	6.8	0.46	0.37
Krill	13	73	86	0.15	0.58
**Bransfield (III)**					
Control	0.8	4.0	4.8	0.16	0.08
NH_4_^+^	0.6	21	22	0.03	0.06
Krill	22	145	167	0.13	0.47

Integrated bacterial production from changes in biomass (BP) and respiration (BR) in the two treatments (+ammonium; +krill excretion products) and controls, along the three experiments. BCD: Bacterial carbon demand (BP+BR). BGE: Bacterial growth efficiency [BP/(BP+BR)]. NGR: Net specific growth rate [ln (BB_T5/_BB_T0_)/T5, being BB bacterial biomass and T time in days].

Additionally, and with the aim of comparing to *in situ* conditions, bacterial production was estimated at the onset of the experiment from ^3^H-Leucine-protein synthesis (BP-Leu), following the microcentrifugation technique proposed by Smith and Azam [26]. Briefly, 5 µl of L-[4,5−^3^H] leucine was added to 1.5 ml replicate samples, yielding a final concentration of 52.7 nM, and was incubated for 2 to 5 h. We used a conversion factor from leucine to carbon incorporation of 1.5 kg C mol leu^–1^, which represents a standard, assuming no isotope dilution [27].

Dissolved organic carbon (DOC) and iron (Fe) were measured only at the onset of the experiments and in the filtered water initially used for all the treatments, respectively, to compare with the *in situ* conditions from where samples were collected (Table 1). Samples for the analysis of DOC were collected after filtration through pre-combusted Whatman GF/F filters into pre-combusted 10 ml glass ampules, acidified with 50 µl of 50% H_3_PO_4_, sealed and stored at 2–4°C until analyzed [28]. DOC concentrations were measured using a Shimadzu TOC-V analyzer. At the beginning of each analysis run, the sample was sparged with CO_2_−free air for several minutes to remove the inorganic carbon. The sample was then injected (3 replicates of 100 µl) into a quartz tube with a platinum catalyst, and combusted at 680°C. DOC concentrations were determined from standard curves (30 to 200 µM C) of potassium hydrogen phthalate produced every day [29]. DOC reference material prepared in the laboratory of Dennis Hansell (Univ. of Miami) was analyzed every day to check for the accuracy and precision of our instrument. Iron concentrations were determined by inductively coupled plasma mass spectrometry (ICP-MS; ThermoFinigan, Element 2) after pre-concentration with amino pyrrolidine dithiocarbamate/diethyl dithiocarbamate (APDC/DDC) organic extraction, as described in Tovar-Sánchez et al. [8].

### Statistical Analysis

Two-way repeated measures ANOVA was applied to look for differences in the response of bacterial populations to different treatments (control, ammonium and krill) over time (T0 to T5) in the three experiments. The primary purpose of the ANOVA was to test if there was an interaction between the factors “time” and “treatments” on the dependent variable (bacterial abundance). Following, post-hoc Bonferroni’s and Tukey’s pairwise means comparisons were performed to find out which specific groups within each factor were significantly different from each other. We used the Mathematica package (v. 9.0) to run the statistical analyses, using a level of significance of 0.01.

## Results

**Figure 2:**
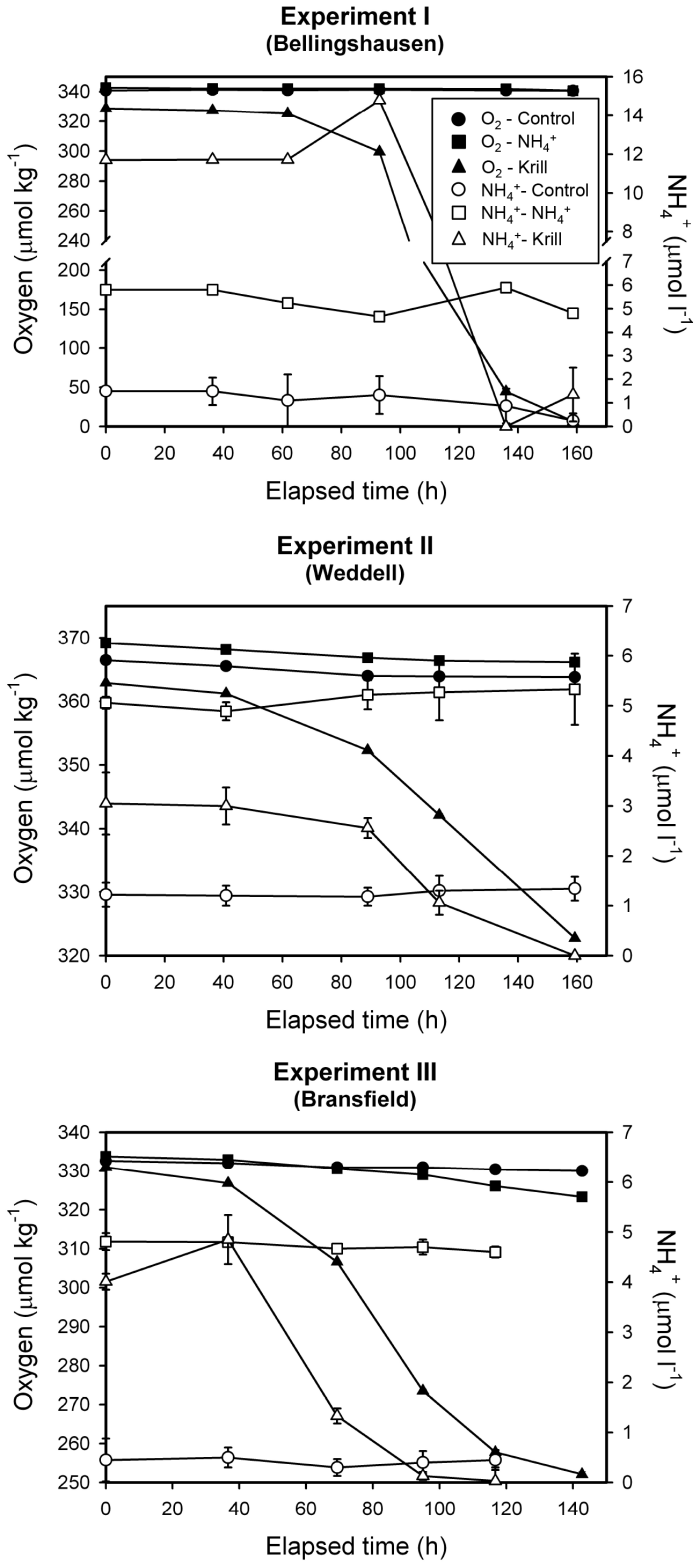
Time series of oxygen and ammonium concentration. Time evolution of DO and NH_4_^+^ in experimental units receiving krill excretion products, ammonium inputs and controls for the three experiments conducted.

At the onset of the experiments, average DOC concentrations in the krill treatments ranged from 188 to 560 µmol C L^−1^, compared to values of 69 to 75 µmol C L^−1^ in control and ammonium-amended treatments, the latter somewhat higher than surface water concentrations measured at the sampled areas during the cruise (Table 1). Ammonium concentrations ranged from 2.6 to 11.7 µmol NH_4_^+^ L^−1^ in the experiments receiving krill excretion products, about 5 µmol NH_4_^+^ L^−1^ in the experiments receiving ammonium inputs, and 0.5 to 1.2 µmol NH_4_^+^ L^−1^ in control treatments (Table 1, Fig. 2). The initial ammonium values in the control treatments were comparable to those measured in the surface waters of the study area (0.1 to 2 µmol NH_4_^+^ L^−1^). Fe levels in the filtered water used for all the experiments and treatments (3–15 nmol Fe L^−1^) were above the ambient levels in krill-free samples from the surface ocean (0.1–0.6 nmol Fe L^−1^), probably due to on-deck contamination after filtering the seawater. The initial Fe concentrations in our experiments were comparable to the daily iron release by krill (0.2–4.3 nmol Fe L^−1^) reported by Tovar-Sánchez et al. [8] during the same cruise, where these authors measured concentrations up to 140 nmol Fe L^−1^ in water samples collected near krill swarms. Hence, we assumed that iron was not a limiting factor for bacterial growth around a krill swarm. Oxygen concentrations at the onset of the experiments varied between 330 and 370 µmol kg^−1^, near or slightly above saturation levels (Fig. 2).

**Figure 3:**
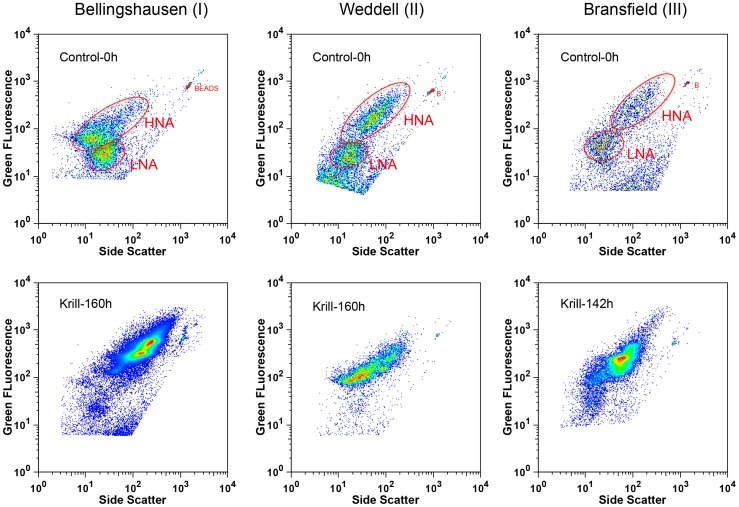
Flow cytometric analysis. Cytograms of bacterial samples at the start of experiments in controls and end of experiments in krill treatments, giving a relative estimate of the distribution of cell groups in each sample. The red lines at the controls broadly separate two groups of bacterial assemblages: (i) HNA: cells with high green fluorescence and large size (side scatter) and (ii) LNA: cells with low green fluorescence and small size (side scatter). Notice that experiments II and III present HNA cells at T0 with higher fluorescence and side scatter than in experiment I (see text for explanation).

Bacterial populations at the start of the experiments (T0) presented differences in abundance, green fluorescence and size (as indicated by side scatter), probably indicating variability in either assemblage composition or cell activity between independent experiments (Table 1, Fig. 3). Bacterial cells were clustered into the commonly used “low nucleic acid (LNA)” and “high nucleic acid (HNA)” groups [30], although assemblages among the HNA groups showed variable side scatter and fluorescence (Fig. 3). Overall, experiments I and II presented lower bacterial abundances at T0 (∼5–9×10^5^ cell ml^−1^) compared to experiment III (∼25–29×10^5^ cell ml^−1^), matching *in situ* abundances (Table 1). However, although in all cases the LNA/HNA ratio was about 1 at T0, experiments II and III showed a significantly higher proportion of cells with larger size and higher fluorescence among the HNA groups (Fig. 3), probably indicating the presence of bacterial assemblages with higher activity. Indeed, average bacterial production (BP, estimated from leucine uptake measurements) at T0 of experiment I was 11 to15 and 43 to 66 times lower compared to experiments III and II, respectively (Table 1). BA and BP estimates from T0 of our experiments were in the same range of values measured from natural populations at the same depths and sites where water was collected (Table 1).

**Figure 4:**
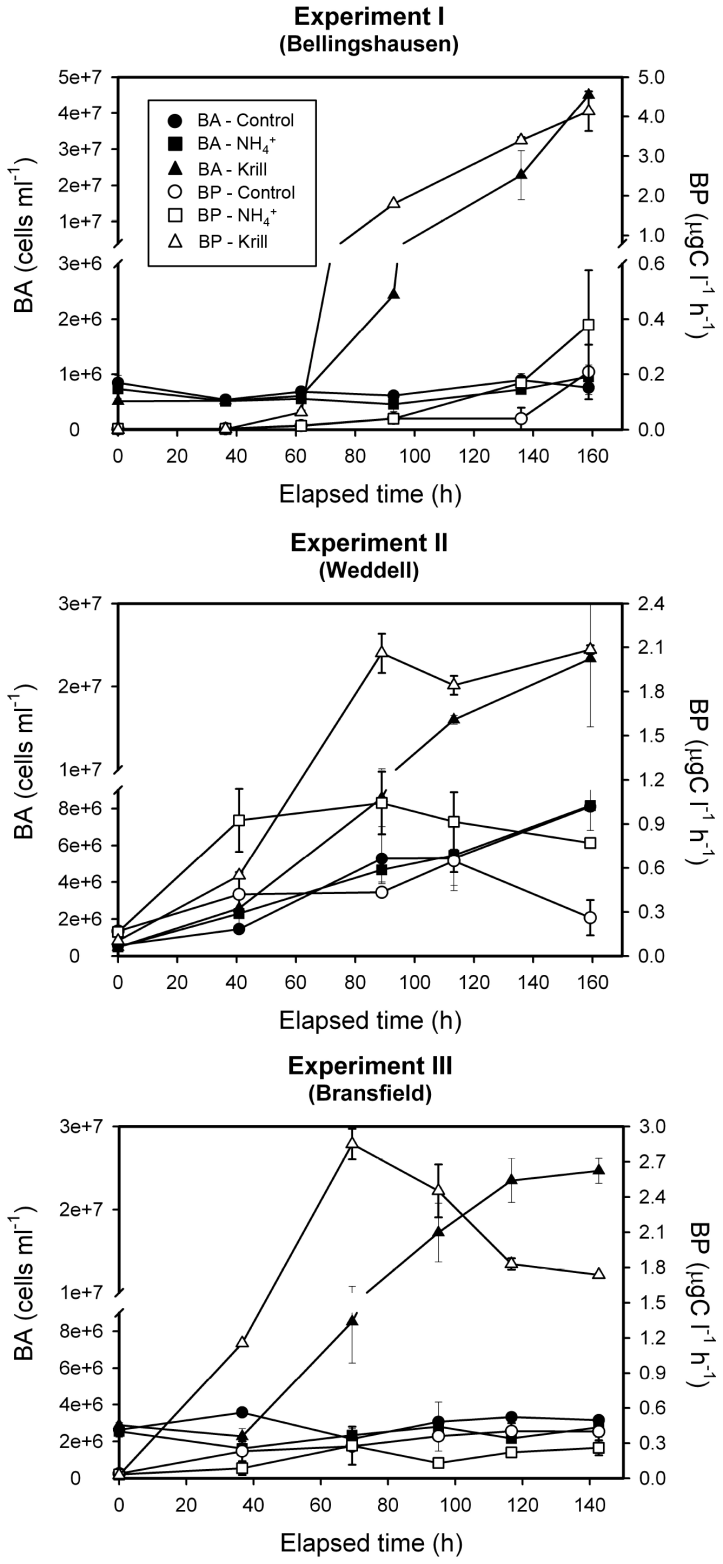
Time series of bacterial abundance. Time evolution of BA in experimental units receiving krill excretion products, ammonium inputs and controls for the three experiments conducted.

**Figure 5:**
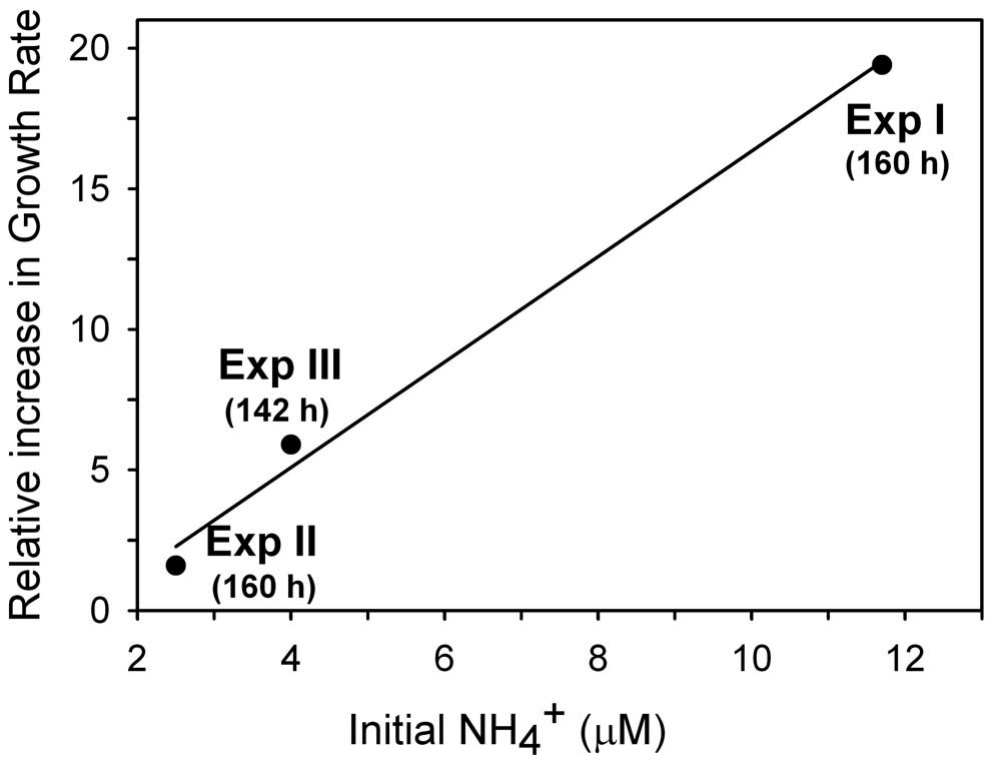
Bacterial growth rate versus NH_4_^+^. The relationship between the ratio of increase in bacterial growth rate in the krill treatments with respect to the controls and the initial ammonium concentration in the treatments receiving krill excretion products, as a proxy of the amount of krill excretion production added.

Variability in the initial populations clearly conditioned the short-term bacterial responses to krill additions between the three experiments. In experiment I, where HNA bacterial cells had lower fluorescence and smaller size at T0, there was a lag period of 3 days, followed by exponential increase in bacterial abundances in the treatment receiving krill products, in spite of a higher addition of organic material and ammonium compared to the other experiments (Table 1 and Fig. 2). In experiments II and III, where HNA cells had higher fluorescence and larger sizes at T0, the lag period was shorter (<2 days in experiment III) or almost absent (<1 day in experiment II). In all cases, however, addition of krill excretion products led to exponential growth of bacterial populations to reach bacterial abundances 10 to 100 fold above the initial values (Fig. 4). In contrast, bacterial populations both in the control and the ammonium treatment behaved identically, either remaining almost stable (experiments I and III) or increasing slightly (experiment II) at levels at least five fold below those in the treatment receiving krill excretion products (Fig. 4). The associated net specific growth rates in terms of biomass (estimated as ln (BB_T5/_BB_T0_)/T5, being BB bacterial biomass and T time in days) increased greatly, to values ranging from 0.47 to 0.97 d^−1^ across experiments, following the addition of krill excretion products, compared to values ranging from 0.05 to 0.08 d^−1^ in control and 0.06 to 0.37 d^−1^ in treatments receiving ammonium inputs alone (Table 2). A strong relationship (r^2^ = 0.99) was found between the increase in growth rates in the treatments receiving krill excretion products relative to the controls and the initial ammonium concentration, as a proxy of the amount of krill excretion production added (Fig. 5), although this relationship should be viewed with some level of caution, since it is based on only three data points.

The responses of bacterial production and respiration to the addition of krill excretion products involved large increases in the carbon flux through bacteria. The calculated bacterial carbon demand (BCD) increased by 14 to 1878 fold in response to the addition of krill excretion products relative to the control, whereas it increased by 1to 10 fold when only ammonium was added (Table 2). However the relative apportioning of carbon use to growth and respiration was not significantly affected by the addition of krill excretion products. Indeed, the bacterial growth efficiency (BGE) was not statistically different between treatments and controls, averaging 0.20 (SE: 0.05), comparable to other reported studies in Antarctic waters, as well as to the global oceanic average [31].

Contrary to ammonium additions, the effect of krill excretion additions on bacterial production (as increase in biomass) was significantly positive in all three experiments, compared to controls and along the time course of the krill experiments. Two-way repeated measures ANOVAs (Table 3) confirm that there were significant differences between treatments and control, time evolution, and their interaction in the three experiments. Post-hoc pairwise mean comparisons, Tukey’s and Bonferroni’s tests, indicate that in all three experiments the bacterial production in treatments receiving krill excretion products differed from that in both the control and ammonium treatments (Table 4). The two post-hoc tests also indicate significant differences in the treatments between the initial times (T0) and the final times (T5). The differences between sampling times were more evident between the time periods T0–T2 and T3–T5, since there was the transition from the latent phase to the exponential growth phase. BP showed larger differences in experiment I, where bacterial biomass increased by 300 times over the control and 378 times over the ammonium treatment (Table 2).

**Table 3:** Statistical ANOVAs analyses.

Experiment	Factor	DF	F - ratio	p - value
**Bellingshausen (I)**	Treatment	2	76.4	<0.0001
	Time	5	20.5	<0.0001
	Interaction	10	15.5	<0.0001
**Weddell (II)**	Treatment	2	128.9	<0.0001
	Time	5	34.1	<0.0001
	Interaction	10	17.2	<0.0001
**Bransfield (III)**	Treatment	2	280.6	<0.0001
	Time	5	40.2	<0.0001
	Interaction	10	21.9	<0.0001

Results of two-way repeated measures ANOVAs to determine the effects of treatment, time evolution, and the interaction between the two factors on bacterial production. The analyses indicate that there are significant differences (p<0.0001) between treatments, time evolution, and their interaction in the three experiments (see text for details). DF = Degree of freedom; F - ratio = Variance ratio.

**Table 4:** Post hoc Bonferroni’s and Tukey’s mean comparisons tests.

Factor	Test	Pair comparisons					
		Bellingshausen Sea		Weddell Sea		Bransfield Strait	
**Treatment**	Bonferroni	(C,K),(A,K)	(C,A),(C,K),(A,K)		(C,K),(A,K)		
	Tukey	(C,K),(A,K)	(C,A),(C,K),(A,K)		(C,K),(A,K)		
**Time**	Bonferroni	(0,3),(0,4),(0,5)	(0,1),(0,2),(0,3),(0,4),(0,5)		(0,2),(0,3),(0,4),(0,5)		
		(1,3),(1,4),(1,5)	(1,2),(1,3)		(1,2),(1,3),(1,4),(1,5)		
		(2,3),(2,4),(2,5)					
	Tukey	(0,3),(0,4),(0,5)	(0,1),(0,2),(0,3),(0,4),(0,5)		(0,2),(0,3),(0,4),(0,5)		
		(1,3),(1,4),(1,5)	(1,2),(1,3)		(1,2),(1,3),(1,4),(1,5)		
		(2,3),(2,4),(2,5)					

Pairwise numbers and letters between brackets indicate that the means of those values were significantly different at p<0.01. Values for “Treatment” are: A = ammonium, C = control, K = krill. Values for “Time” are 0 = T0, 1 = T1,… 5 = T5 (see text for details).

The addition of krill excretion products also greatly stimulated respiration rates, compared with the ammonium treatments and controls (Tables 2 and 3). In experiment I, where the added excreted ammonium and organic carbon was highest, the effect was so large that oxygen concentrations in the experimental units, which were not open to the atmosphere, declined to hypoxic conditions (4 µM O_2_ compared to an initial value of 328 µM O_2_) 160 hours following the onset of the experiment (Fig. 2).

## Discussion

The results of our experiments demonstrated very large effects of krill excretion products on bacterial growth, production and respiration, in spite of the variability in the initial conditions of the experiments. In general, direct ammonium inputs produced moderate increases in bacterial production and respiration rates (except in experiment II), which were non significant compared to controls (Tables 2–4). However, the addition of krill excretion products greatly stimulated heterotrophic bacteria far beyond the effect of ammonium inputs alone, demonstrating that the role of krill excretion in stimulating bacteria is not directly connected to their excretion of ammonium. Indeed, earlier studies demonstrated that bacterial growth in polar regions is mainly controlled by organic matter supply [10], [11], which limits the ammonium uptake by bacteria [32]. Church et al. [11] also suggested that bacterial growth could be co-limited by iron availability in the Southern Ocean. In our experiments, the iron levels at the onset of the experiments were higher than the ambient levels in the regions of study, but were lower than in *in situ* water samples collected at stations with presence of krill swarms [8]. Hence, it is unlikely that iron was limiting bacterial growth in our experiments, as it would not limit bacterial growth in the field after the passage of a krill swarm.

Observations of greatly increased bacterial remineralisation rates in areas with high krill activity led Goeyens et al. [33] to suggest that krill swarms generate large amounts of organic substrates when grazing on autotrophic and heterotrophic prey, triggering bacterial activity. Supporting this hypothesis, Ruiz-Halpern et al. [17] recently estimated, through a series of experiments in the Antarctic Peninsula, that krill supplied on average 150 mmol DOC m^−2^ d^−1^, contributing about 75% of the combined krill - phytoplankton production of DOC in this Southern Ocean ecosystem. In addition, Ortega-Retuerta et al. [16] showed that in hourly incubations the optical signatures of krill excretion with marked peaks in short wavelengths (250–300 nm) were similar to spectroscopic scans for aromatic amino acids such as tryptophan, tyrosine, or phenylalanine [34], a probable high quality substrate for bacteria. The experimental results reported here conclusively show that krill excretion products greatly enhance bacterial growth and metabolism.

There are, however, caveats in extrapolating our results to the field, as the interaction between krill and bacteria would depend on the time bacteria assemblages remain in contact with a krill swarm and the diffusion rates of krill excretion products in the water. Krill spend most of their 5–7 year lifetime in swarms of different size, depending on the maturity status of the individuals [19], and may eventually reach “super-aggregations” over areas of hundreds of km^2^ at densities of >1000 individuals m^−3^ [18], [19] Moreover, they would also constitute extended regions of high (organic and inorganic) nutrient availability.

Our experimental design was intended to simulate bacterial responses to large krill swarms excreting over about several hours in a same region. Stocker et al. [35] demonstrated that some bacteria could efficiently exploit ephemeral nutrient patches, resulting from sloppy feeding or organic matter release, before physical mechanisms dissipate them, by using chemotactic swimming strategies. The fast response time scales of these “opportunistic” bacteria would enable them to utilize a wide range of patchily distributed nutrient resources in the ocean [35]. The cytometric analysis of our samples (Fig. 3) shows two broad groups of bacteria assemblages at the onset of the experiments: (LNA) low fluorescence and small size cells (presumably with small genomes), and (HNA) high fluorescence and large size cells (with large genomes). The shortest delay in response to krill excretion products is observed in experiments II and III, where the HNA cells present higher side scatter and fluorescence than in experiment I. These cells are clearly dominant at the end of the three experiments (Fig. 3). Lauro et al. [36] argued that “copiotrophic” bacteria (growing at high nutrient concentrations) have higher genetic potential to sense and rapidly respond to sudden nutrient influx, compared to the more widely distributed “oligotrophic” bacteria (growing at low nutrient concentrations) which dominate the ocean’s free-living microbial populations. “Copiotrophs”, like our HNA bacteria, are large (>1 um in diameter), grow rapidly (doubling times of hours) and carry relatively large genomes, which allow them to evolve complex systems for sensing and responding to sudden changes in their environment [36], [37]. Our results suggest that the larger HNA bacteria of our experiments, which showed a dramatic response to krill excreta additions, may correspond to the “opportunistic” and “copiotrophic” forms described, respectively, by Stocker et al. [35] and Lauro et al. [36].

Adults and larval stages of krill feed not only on phytoplankton but also on heterotrophic protozoans (like ciliates), releasing grazing pressure on microbial communities [38]. Goeyens et al. [33] reported an extremely high bacterial activity and almost negligible protozoan grazing after the recent passage of a krill swarm, at one station in the Scotia-Weddell Confluence Area. While feeding on bacterial grazers, krill release large amounts of organic (DOM) and inorganic (ammonium, phoshate, iron, etc.) nutrients [8], [17], [20], [39] creating optimal conditions for the exponential growth of bacteria, as demonstrated in our experiments. Increased remineralization by bacteria would lead to enhanced availability of ammonium [40], [41], fuelling phytoplankton growth, once the krill grazing pressure is released [42].

Krill may exhibit a “superfluous feeding” behaviour, which leads to deplete large phytoplankton blooms together with ciliates in a few hours [43], [44], [45]. Intense grazing by krill leads to high recycling by krill themselves as well as bacteria stimulated by krill excretion products, minimising sinking losses of nutrients. The immediate consequence of this “krill-bacteria recycling loop” would be an enhanced remineralization of the dissolved organic matter released by krill. Smetaceck [7] suggests that bacterial remineralization of krill excretion products is a highly efficient mechanism to increase the residence time of iron in the surface ocean, thereby conditioning the environment to support high phytoplankton growth. Nevertheless, both experimental studies [38], [46] and field observations [47], indicate that it is the small nanophytoplankton (not preyed by krill) that would take advantage of the krill-excreted and bacteria-remineralized nutrients in the water column, leading to the development of blooms. Thus, as Smetacek [7] argues, the overall effect would be to enhance the recycling of nutrients (including iron) in the surface ocean. In the context of the biological pump, an increased remineralization of organic matter by bacteria in the surface ocean would lower the efficiency of the ecosystem to export carbon to the deep-ocean. However, it would also maintain iron available at surface to be used by phytoplankton and bacteria [11], supporting further blooms development. Faecal pellets are probably the dominant component of carbon export in regions where krill are abundant [15]. However, krill also contribute largely to the excretion of dissolved organic products [16], [17]. Our results give support to the hypothesis of an efficient recycling of these products by bacteria and enhanced respiration rates. This recycling system would lead to a loss of CO_2_ to the atmosphere, but at the same time would trigger the formation of new blooms, which might eventually be grazed by zooplankton or sink down to the deep ocean.

In addition to highlighting the role of krill in stimulating bacterial growth and activity, the experimental results reported here provide insights into the constraints to bacterial growth in the Southern Ocean. Bacterial growth rates are remarkably low, and weakly coupled to primary production in the Southern Ocean [12]. The results presented here suggest that the maximum growth and metabolic rates possible for bacteria in the cold waters of the Southern Ocean are comparable to those in warm waters, and that the realised growth rates are kept low due not to low temperature but to resource limitation, providing no grazing pressure [48]. Nevertheless, low temperature affects the structure of bacterial membranes, which become gel-like and more resistant to diffusive flow of substrates [49], and may limit bacterial growth by enhancing their substrate requirements [49], [50], [51]. These arguments are consistent with our results, as an addition of substrates released by krill, including inorganic and organic nutrients, enhanced bacterial growth by a factor of 10 over ambient values.

Obviously, our results on bacterial metabolic activity are conditioned by the experimental design, and cannot be readily extrapolated to the field. The highest rates of BP measured in our experiments exceeded several folds the maximum rates measured in the field in polar waters [31]. The release of grazing pressure by selective filtration prior to our experiments would have allowed the largest and more active bacterial cells to grow with the only limitation of the available substrates. Growing in small incubation bottles might have also stimulated bacterial activity by the “wall-effect”. Even pre-filtration of the bacteria cultures before incubation could be another possible source of labile DOM, large enough to slightly stimulate bacterial growth. All this could explain that even untreated controls exceeded in some cases, after several days of incubation, the BP rates measured in the field, but cannot account for the huge difference in response between controls and treatments receiving krill excretion products.

Hence, the potential constraints do not invalidate our main conclusions. The results presented here confirm that the addition of krill excretion products (but not of ammonium alone), at levels expected in krill swarms, greatly stimulates Southern Ocean bacteria (in the absence of predation) resulting in order-of-magnitude increase in growth and production, compared to untreated samples. These results suggest that bacterial growth rate in the Southern Ocean is suppressed well below their potential by acute resource limitation. Enhanced bacterial activity in the presence of krill, which are major sources of DOC in the Southern Ocean further increases recycling processes associated with krill activity, resulting in a highly efficient krill-bacterial recycling that should be conducive to stimulating periods of high productivity in the Southern Ocean.
